# Supplementary material for: Seroreactivity to Coxiella burnetii in an Agricultural Population and Prevalence of Coxiella burnetii Infection in Ticks of a Non-Endemic Region for Q Fever in South Korea
Source: Pathogens. 2021 Oct 17;10(10):1337. doi: 10.3390/pathogens10101337 (PMC8538241; doi:10.3390/pathogens10101337)
Supplement: Supplementary file 1 [file pathogens-10-01337-s001.zip › pathogens-1337633-SI.pdf]

Supplementary Table S1. Coxiella burnetii detection and developmental stages of 354 collected ticks by every months in Jeju Island (2016-2018).

	JJ		AW		HD		BD		SH		Total	
	Tick*	***C. burnetii*** **(IR, %)**	Tick*	***C. burnetii*** **(IR, %)**	Tick*	***C. burnetii*** **(IR, %)**	Tick*	***C. burnetii*** **(IR, %)**	Tick*	***C. burnetii*** **(IR, %)**	Tick*	***C. burnetii*** **(IR, %)**
**Adult (F)**	3/51	**0/3 (0)**	13/73	**0/13 (0)**	15/76	**0/15 (0)**	4/55	**0/4 (0)**	15/98	**2/15 (13.3)**	**50/353**	**2/50 (4)**
**Adult (M)**	3/25	**0/3 (0)**	7/52	**0/7 (0)**	9/52	**1/9 (11.1)**	0/32	**N/A**	13/80	**0/13 (0)**	**32/241**	**1/32 (3.1)**
**Nymph**	40/476	**4/40 (10)**	70/578	**3/70 (4.3)**	61/583	**3/61 (4.9)**	31/362	**3/31 (9.7)**	70/598	**3/70 (4.3)**	**272/2,597**	**16/272 (5.9)**
**Larvae**	0	**N/A**	0	**N/A**	0	**N/A**	0/1	**N/A**	0	**N/A**	**0/1**	**N/A**
**Total**	46/552	**4/46 (8.7)**	90/703	**3/90 (3.3)**	85/711	**4/85 (4.7)**	35/450	**3/35 (8.6)**	98/777	**5/98 (5.1)**	**354/3,193**	**19/354 (5.4)**

C. burnetii, Coxiella burnetii; JJ, Jeo Ji; Aewol (AW); Seon Hul (SH); Ha Do (HD) and ; Bo Mok-ri (BM); IR, infection rate; F, female; M, male; N/A not applicable**.** * Of 3,193 ticks collected in this study periods, clone XCP-1 16S ribosomal RNA gene sequencing was examined to identify *Coxiella* species in the 354 ticks. Of total collected ticks, *Haemaphysalis longicornis* was detected in every month, and *Haemaphysalis flava* was detected in January, Febrary, April and November. The *C. burnetii*-infected ticks were detected only in *Haemaphysalis longicornis.*
